# Impregnation of High-Magnetization FeCo Nanoparticles in Mesoporous Silicon: An Experimental Approach

**DOI:** 10.3389/fchem.2018.00609

**Published:** 2018-12-18

**Authors:** Mathieu Lepesant, Benjamin Bardet, Lise-Marie Lacroix, Pierre Fau, Cyril Garnero, Bruno Chaudret, Katerina Soulantica, Thomas Defforge, Damien Valente, Caroline Andreazza, Jérôme Billoué, Patrick Poveda, Gaël Gautier

**Affiliations:** ^1^LCC-CNRS, Laboratoire de Chimie de Coordination, CNRS, UPS, Toulouse, France; ^2^Laboratoire de Physique et Chimie de Nano-Objets, UMR 5215 INSA-CNRS-UPS, Université de Toulouse, Toulouse, France; ^3^Groupe de Recherche en Matériaux, Microélectronique, Acoustique et Nanotechnologies, UMR CNRS 7347, INSA-CVL, Université de Tours, Tours, France; ^4^ST Microelectronics, Tours, France; ^5^Interfaces, Confinement, Matériaux et Nanostructures, CNRS, UMR 7374, Université d’Orléans, Orléans, France

**Keywords:** impregnation, magnetic nanoparticles, mesoporous silicon, nanocomposite, magnetic properties

## Abstract

This paper deals with the synthesis of high-magnetization porous silicon-based nanocomposites. Using well-controlled organometallic synthesis of ferromagnetic FeCo nanoparticles, the impregnation of mesoporous silicon has been performed by immersion of porous silicon in a colloidal solution. The technique was optimized by controlling the temperature, the immersion duration, and the solvent nature. The characterization of the nanocomposites showed a homogeneous filling of the pores and a high magnetization of 135 emu/cm^3^. Such composites present a great interest for many applications including data storage, medical instrumentations, catalysis, or electronics.

## Introduction

Magnetic materials such as iron alloys or iron oxides (FePt, FeCo, Fe_2_O_3_, etc.) present a great interest in data storage (Sun et al., 2000), drug delivery (Anglin et al., 2008; Zhu et al., 2009), medical instruments (ex: for magnetic resonance imaging; Bomati-Miguel et al., 2005; Thomas et al., 2006) or catalysis (Manova et al., 2006; Li et al., 2011; Bordet et al., 2016). For instance, the Fischer–Tropsch process is a widely studied method that uses magnetic metals such as iron or cobalt as catalysts to produce synthetic fuel (Bordet et al., 2016). Due the progressive disappearance of fossil fuels, this catalysis process appears nowadays as an attractive way to produce “green” synthetic fuels from biomass or natural gas sources. More specifically, employment of nanosized magnetic materials show an improved reactive surface area and their insertion in a porous medium is one of the solutions to prevent their mutual aggregation and sintering during reaction (Lu et al., 2004; Bomati-Miguel et al., 2005). Porous substrates made of different materials such as PEGDA (polyethylene glycol diacrylate; Allia et al., 2011), silicon (Anglin et al., 2008; Granitzer et al., 2010; Harraz, 2013) or silica (Lu et al., 2004; Nakamura et al., 2006; Kockrick et al., 2007; Zhu et al., 2009; Liu et al., 2011; Kim et al., 2013) can be used as supports. In particular, porous silicon (PS) is one of the most interesting media because it is a versatile material with tunable surface chemistry, pore size or surface area (Loni et al., 2015) and shows interesting behavior concerning biodegradability issues (Xia et al., 2017). However, the methods employed to fill such porous material with nanoparticles (NPs) remain very challenging. Two strategies of filling are reported in the literature. In the case of *in situ* synthesis techniques, precursors are introduced by impregnation within the porous template and then directly reduced. However, this technique suffers from a poor renewing of precursors, a low in-depth homogeneity and strongly depends on silicon surface chemistry (Yiu et al., 2008; Fukami et al., 2009; Bardet et al., 2017). Another technique consists in impregnating the porous material with *ex situ* synthetized and stabilized NPs. In this way, a better control of the NP properties is achieved (Granitzer et al., 2013, 2015; Rumpf et al., 2013). However, the influence of the *ex situ* loading with regard to previous particle synthesis has not been widely studied and reported in the literature.

In this paper, we propose to load FeCo magnetic NPs with a regular cubic geometry into mesoporous silicon. This study aims at providing an experimental procedure to impregnate PS to high loadings and to characterize the in-depth homogeneity and the magnetic properties of the nanocomposite.

## Experimental

### Synthesis of FeCo Nanoparticles

FeCo NPs were synthesized by adaptation of a previously reported organometallic approach (Lacroix et al., 2009). Depending on the synthesis conditions, the size of the particles can be varied from 5 to 15 nm. In this work, we prepared FeCo NPs of 9.0 ± 0.6 nm. Fe and Co silylamides, (- Fe(N(Si(CH3)_3_)_2_)_2_)_2_ and Co(N(Si(CH_3_)_3_)_2_)_2_(THF) -) were reduced under 3 bars of H_2_ at 150°C for 48 h in mesitylene and in the presence of long chain surfactants (hexadecylamine—HDA, and palmitic acid—PA). The precursors concentrations were kept at 20 mmol/L. The HDA and PA concentrations were 80 and 55 mmol/L, respectively. After reaction, the excess of surfactant was removed by magnetically-assisted separation under inert atmosphere to prevent any oxidation. The particles were then kept as powder in the glove box. Chemical analyses were performed on FeCo NPs by inductively coupled plasma mass spectrometry (ICP-MS) revealing a global composition of Fe_52_Co_48_.

### Synthesis of Porous Silicon

Porous silicon templates were synthesized by anodization of a highly-doped n-type silicon wafer with a resistivity of 10–20 mΩ.cm in an electrochemical cell containing concentrated hydrofluoric acid diluted in water (5 vol. %) and Triton X-100 (0.2 mmol/L). A current density of 25 mA/cm^2^ was applied in order to form large and straight pores (Harraz, 2013). The anodization duration was adapted in order to form a PS layer with a thickness of 18 μm. An average pore diameter of 100 nm was estimated by Scanning Electron Microscopy (SEM). A mean porosity (ratio of pore volume and overall volume) of around 66% was calculated from optical FTIR (Fourier Transform Infra-Red spectroscopy) measurements. After the anodization, the samples were thoroughly rinsed in ultrapure water and dried on a hot plate at 120°C. PS wafers were then singulated in 4 × 4 mm^2^ square dices.

### Impregnation Technique

The magnetic cubic NPs were loaded in the PS template by a simple immersion of the substrate in a FeCo colloidal solution at a concentration of 1.3 g/l. The setup is quite similar to the one proposed by Granitzer et al. (2013). However, the role of the solvent and the impregnation temperature is highlighted in this study and will be addressed in the next sections. After impregnation, the substrates were washed with a flow of tetrahydrofuran (THF) in order to eliminate most of the NPs aggregates left on the surface.

### Structural and Morphological Characterization

The particles were characterized by SEM and Transmission Electron Spectroscopy (TEM), using a 100 kV Jeol JEM 1011 and a 200 kV FEI CM20. In order to characterize the particles, they were re-dispersed in toluene and a drop was deposited on a carbon coated copper grid. The nanocomposites were also characterized by Energy Dispersive X-Ray Spectroscopy (EDX) in order to estimate the in-depth homogeneity of the pore filling. Cross section samples were also prepared in order to observe by TEM the NPs impregnation along the PS pores.

### Magnetic Characterization

Magnetic measurements were performed using a Quantum Design Physical Property Measurement System (PPMS) with a Vibrating Sample Magnetometer (VSM). PS substrates filled with NPs were characterized after immobilization on a quartz support using adhesive Kapton film. Hysteresis loops were recorded at 300 and 5 K applying induction field of ±3T. The exchange bias *H*_*ex*_ was determined from the hysteresis loop recorded at 5 K after cooling the sample from 300 K down to 5 K under an external field of 3T.

(1) $$\begin{array}{l} H_{ex}=\frac{H_{c}^{-}+H_{C}^{+}}{2} \end{array}$$

where *H*_*c*_^−^ is the coercive field observed during the demagnetization process (second quadrant, experimentally: −125 mT) and *H*_*c*_^+^ the coercive field observed during the magnetization (fourth quadrant, experimentally: 126 mT).

The pore filling factor Q, which characterizes the mass of NP loaded over a given surface area, was determined following Equation (2):

(2) $$\begin{array}{l} Q\;\left(g/m^{2}\right)=\;\frac{Ms}{\Gamma \times S} \end{array}$$

With Γ the saturation magnetization of the NPs (224 emu/g_FeCo_), Ms the saturation magnetization of the composite determined experimentally as the magnetization at 3T, and S the footprint area of the substrate, i.e., 16 mm^2^.

## Results

### Material Structural Characterization

**Figure 1:**
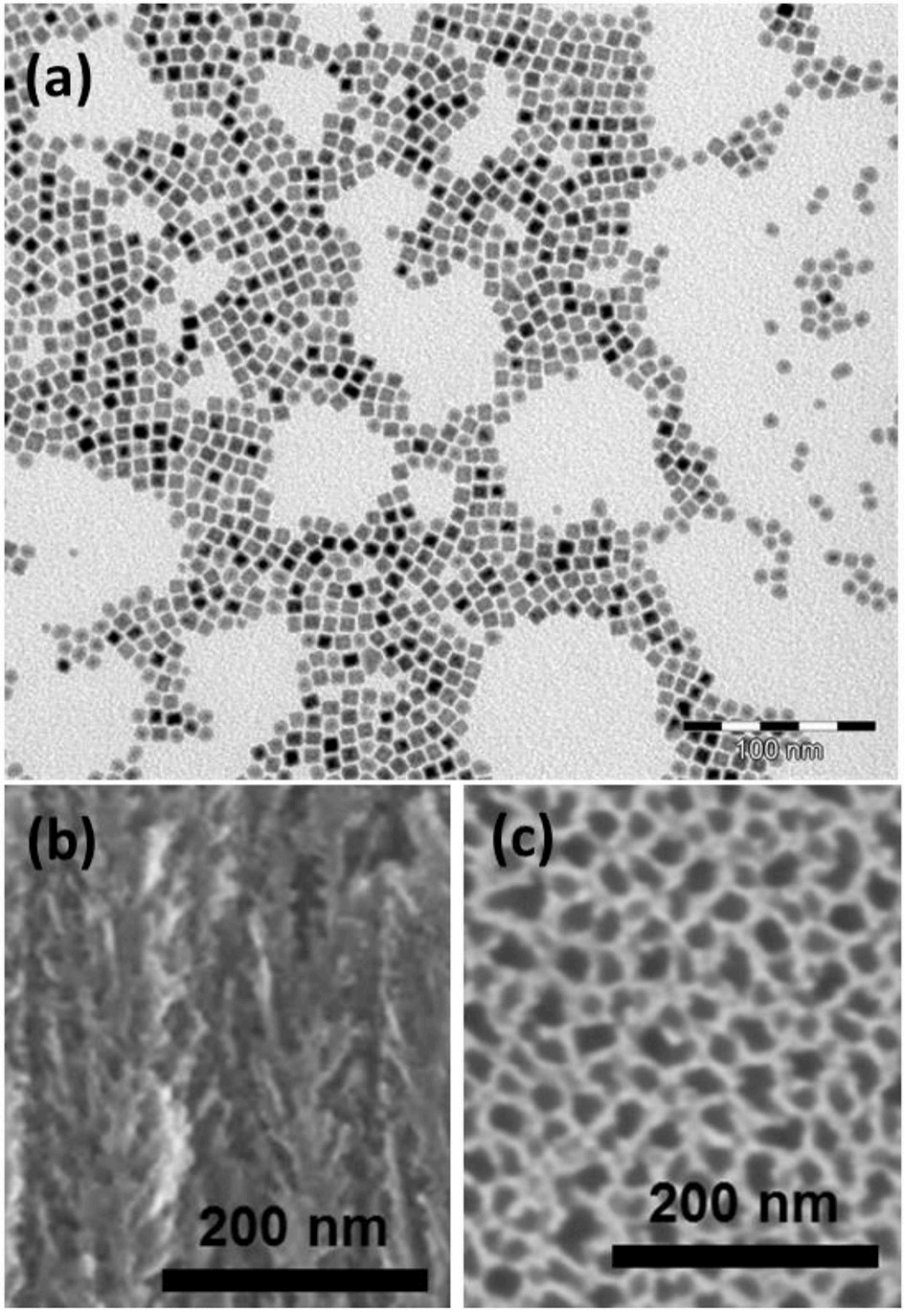
**(a)** TEM image of the as-prepared cubic FeCo nanoparticles; **(b)** cross section and **(c)** top view SEM images of PS.

FeCo NPs were prepared following an organometallic approach. The Co and Fe precursors were decomposed in presence of dihydrogene leading to monodisperse NPs exhibiting a cubic shape as shown in Figure 1a. The particle mean size of 9.0 ± 0.6 nm was well controlled thanks to the stabilization of the surface with a mixture of carboxylic acid and alkylamine ligands. The particles displayed a magnetization at high field (3T) of 204 emu/g_FeCo_ and 217 emu/g_FeCo_, at 300 and 5 K, respectively, both values being close to the bulk saturation magnetization (230 emu/g_FeCo_, Figure S1). The particles are not oxidized as revealed by the absence of any exchange bias at low temperature. This result was further confirmed by Mössbauer spectroscopy (Garnero, 2016).

The PS substrate was also characterized by electron microscopy. The Figures 1b,c show the PS layer before the NPs impregnation. The thickness of the PS layer is about 18 μm. As we can observe in the Figure 1b, the structure is composed of primary pores oriented perpendicularly to the surface with a size in the range of 25–30 nm and secondary smaller branches which is typical of mesoporous silicon etched from highly doped wafers (Garnero, 2016). It is assumed that this secondary porosity is susceptible to bring an improved anchoring surface for the NPs. It is noteworthy that mesoporous silicon obtained by electrochemical etching produces non-interconnected pores (Lehmann et al., 2000). In other words, the only direction for particle impregnation in each pore is from the surface toward the PS/silicon interface.

### Optimization of Pore Filling

**Figure 2:**
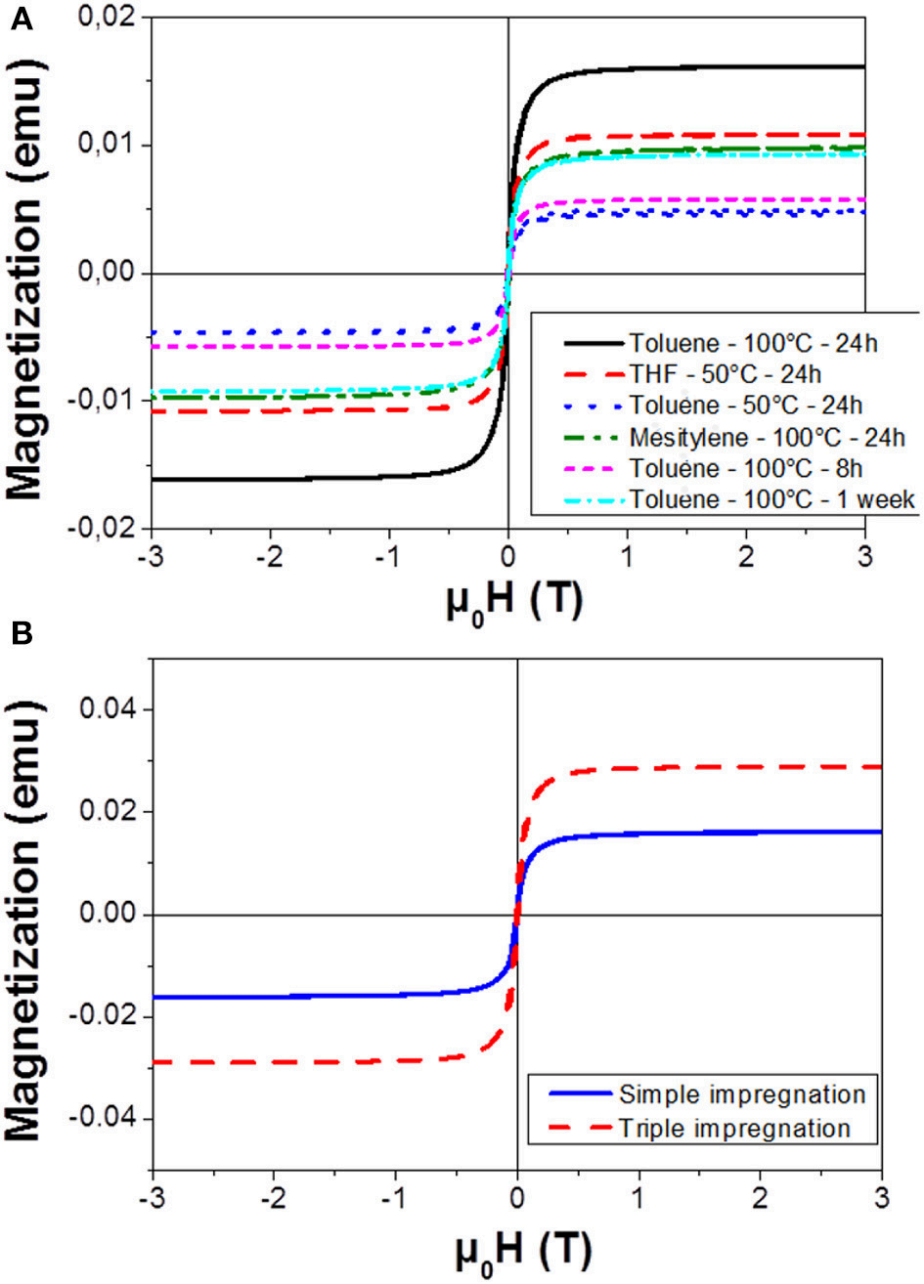
Magnetization curves recorded at 300 K for NPs impregnated into PS with **(A)** different durations, temperature and solvents and **(B)** with a repetition of the impregnation technique (24 h in Toluene at 100°C).

In this paper, we investigate the impact of multiple parameters: the temperature of the colloidal solution, the nature of the solvent and the impregnation duration. The pore filling factor Q, calculated from the magnetic response of the filled PS (Figure 2), is shown in Table 1.

**Table 1:** Summary of saturation magnetization (Ms) and pore filling factor (Q) for different experimental conditions of Figure 2.

**Duration**	**24 h**				**8 h**	**1 week**	**3 × 24 h**
Temperature (°C)	50		100		100	100	100
Solvent	Toluene	THF	Toluene	Mesitylene	Toluene	Toluene	Toluene
M_S_ (memu)	4.9	10.8	16.2	9.8	5.8	12.6	38,9
Filling Factor Q (g/m^2^)	1.4	3.0	4.5	2.7	1.6	2.6	10,9

Impregnations were initially carried out using toluene as solvent, keeping the impregnation duration constant at 24 h. Increasing the temperature of the colloidal solution from 50 to 100°C allowed improving the filling of the pores as revealed by the higher saturation magnetization obtained. This phenomenon can be attributed to a better diffusion of the particles along the pores.

In order to study the influence of the solvent, we compared the magnetic properties of the filled PS using mesitylene (previously used during NPs synthesis) and THF, which is also a good solvent for the colloidal dispersion of the FeCo NPs. As observed in Figure 2A, the impregnation in toluene gave the best overall result. The superiority of toluene compared to mesitylene could be explained by a lower viscosity of the former, 0.59 and 0.66 cP at 25°C, respectively (Rumpf et al., 2013). The THF is a good solvent for NPs dispersion and moreover, it presents the lowest viscosity of all tested solvents (0.55 cP at 25°C). It shows a better impregnation performance than toluene at the low temperature (50°C). However, a low boiling point (66°C) limits the maximum temperature reachable, and consequently the diffusion of the particles. The impregnation into the pores was therefore reduced compared to the results obtained at 100°C in toluene.

The experimental condition of solvent and temperature being optimized, the effect of impregnation duration was studied from 8 h to 1 week. Eight hours of contact was not a sufficient duration to fully fill the pores, as revealed by the low saturation magnetization and the limited filling factor (Q = 1.6 g/m^2^). After a 24 h impregnation, a filling factor of 4.5 g/m^2^ was reached. Extending further the impregnation from 24 h up to a week did not promote a better loading (Figure S2). On the contrary, a decrease of the Q factor was observed following a non-monotonic trend, probably due to a dynamic process of release of the NPs initially hanged on the PS pores back to the colloidal solution. As a result, the best impregnation condition was obtained after an impregnation of 24 h in toluene at 100°C.

As it was not possible to increase the impregnation duration without a loss of NP material, we experimented the repetition of the impregnation procedure three times in the same porous host sample. We observe on Figure 2B an increase of the saturation magnetization and thus of the filling factor, after these three consecutive impregnations. This result highlights the benefit of the re-introduction of fresh NPs within the solution in order to improve the PS filling.

### Structural Characterization of the Nanocomposite

**Figure 3:**
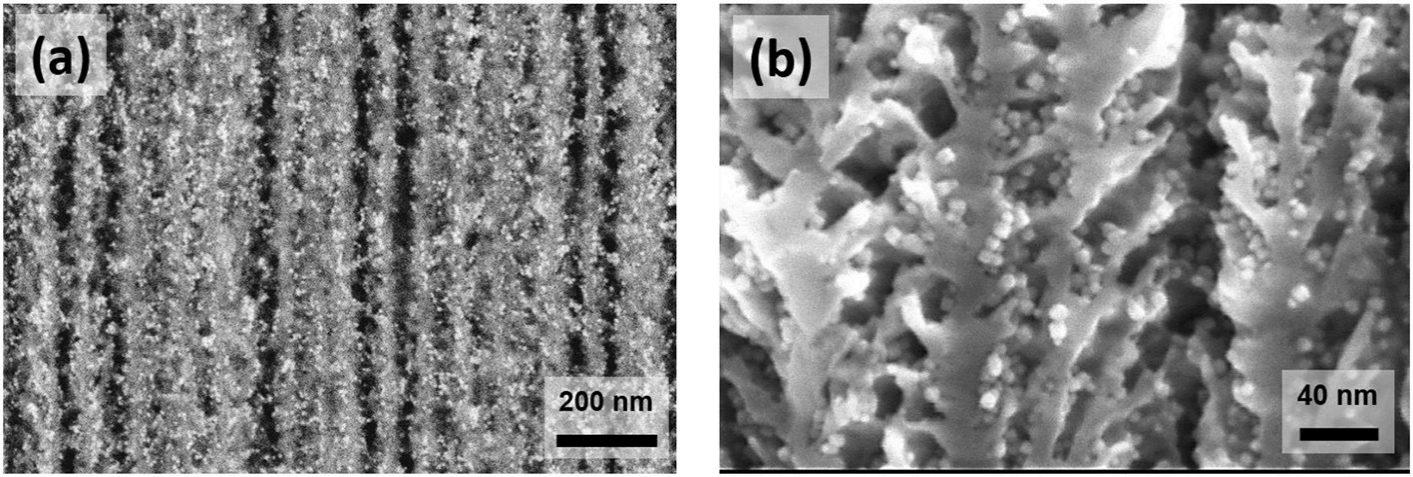
SEM images of PS/FeCo nanocomposites observed with back-scattered electrons detector **(a)**, and with secondary electrons detector **(b)**.

**Figure 4:**
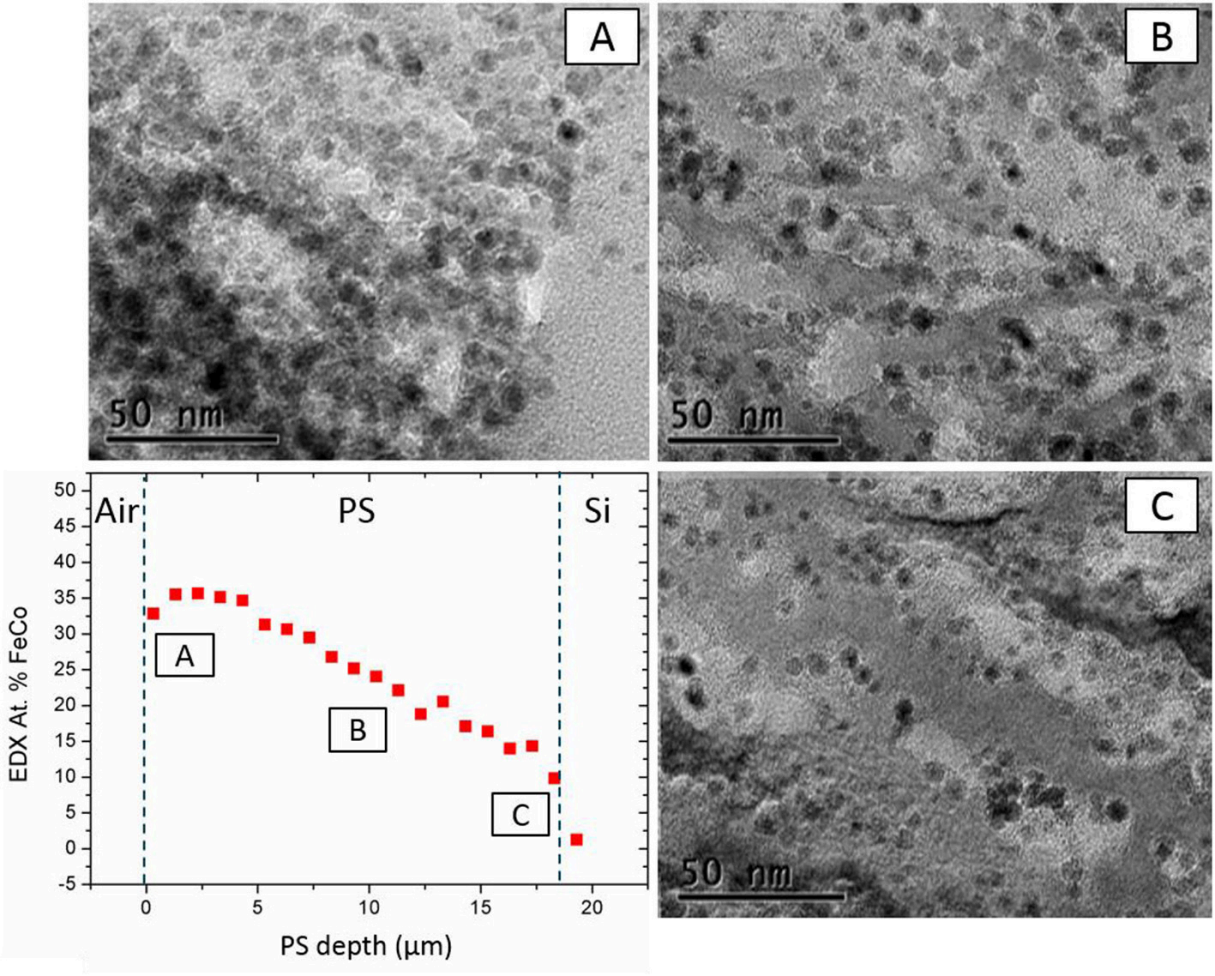
TEM images at different depths: near the Air/PS interface **(A)**, at the middle of the PS layer **(B)**, and near the PS/Si interface **(C)** in perspective with FeCo at % (EDX) in-depth profile.

SEM associated to EDX, TEM, and VSM techniques were employed to characterize the PS impregnated under optimized conditions. SEM images of the impregnated substrate are shown in the Figures 3a,b. These pictures show a cross section of impregnated PS, the white spots corresponding to FeCo NPs (electron back-scattering). As we can see, these nanoparticles are homogeneously dispersed along the pores. The homogeneity of the filling was evaluated by the EDX measurements correlated with TEM micrographs taken at different depths and presented in the Figure 4. In the graph (Figure 4 bottom left), we estimate the atomic percentage of iron and cobalt at different depths from the PS surface down to the Si/PS interface. The results show a quite good homogeneity of the NPs filling, with metal loading from 35% near the PS surface to 15% at the interface. A slight increase of the amount of FeCo is evidenced at the very near surface. This phenomenon can be attributed to the variation of PS morphology with the depth. The pores are indeed less branched at the Si/PS interface, thus leading to lower local specific surface area and less NP anchorage sites. The EDX analysis was confirmed by TEM imaging at different depths (cf. Figures 4A–C) showing a filling along the entire PS pores length. The slight modification of the NPs shape, from cubic like to spheroid, could be attributed to the extended oxidation induced by the TEM sample preparation.

**Figure 5:**
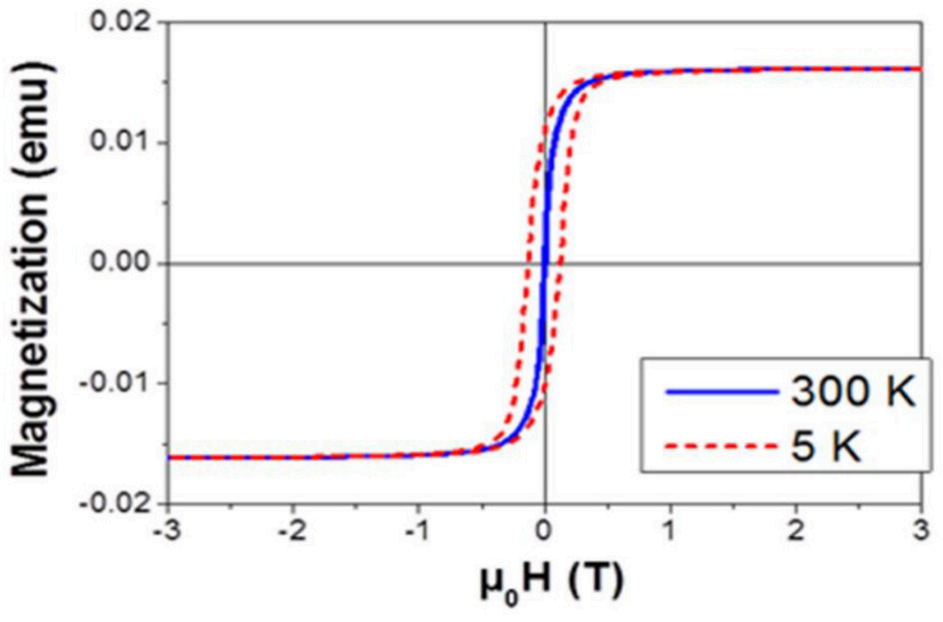
Magnetization curves of the filled PS at 300 K (blue solid line) and 5 K (dashed red line).

The magnetization curves have been obtained by VSM measurements performed at two temperatures, 300 and 5 K (Figure 5). The room temperature measurement allows confirming the filling factor at 10.9 g/m^2^ from the saturation magnetization. The low temperature hysteresis curve was recorded after a 3T field cooling procedure. At 5 K, the NPs appear to be in a blocked state, as revealed by the appearance of a coercive field. The hysteresis loop being symmetric, no exchange bias was detected. One can therefore conclude that the FeCo NPs remained purely metallic, the impregnation technique and the adsorption into PS did not lead to the oxidation of the highly sensitive NPs.

## Conclusion

In this paper, we report the *ex-situ* impregnation of 9 nm FeCo nanoparticles in an 18 μm-thick mesoporous silicon substrate with a pore diameter of about 30 nm. The optimization of the impregnation process parameters allowed defining the optimal conditions to get the best filling factor of the pores and the highest magnetization level. Toluene, heated up to a temperature of 100°C, was found to be the most suitable solvent. Furthermore, an impregnation duration of 24 h was assessed in order to obtain the amount of NPs in the substrate. This step can be repeated several times with fresh colloidal solutions in order to increase the density of nanoparticles embedded in the material. A maximum saturation magnetization around 39 memu (135 emu/cm^3^) is obtained when the impregnation is repeated 3 times with an intermediate rinsing and drying step. We have characterized these samples by electron microscopy (SEM, TEM, and EDX) and found evidences of a high homogeneity of filling in depth. Finally, we showed that these nanoparticles do not exhibit significant oxidation after impregnation thus retaining their metallic nature and magnetic properties for future applications. The next step of this study will be to upscale these experiments on larger surfaces in order to bring this new composite material toward industrial applications.

## Author Contributions

JB, PP and GG designed the project and performed the overall supervisions of the experimental work. All authors discussed the results and revised the manuscript.

### Conflict of Interest Statement

The authors declare that the research was conducted in the absence of any commercial or financial relationships that could be construed as a potential conflict of interest.
