# Supplementary material for: Impregnation of High-Magnetization FeCo Nanoparticles in Mesoporous Silicon: An Experimental Approach
Source: Front Chem. 2018 Dec 18;6:609. doi: 10.3389/fchem.2018.00609 (PMC6305395; doi:10.3389/fchem.2018.00609)
Supplement: Supplementary file 1 [file Table_1.DOCX]

**Supplementary Information**

**Impregnation of high-magnetization FeCo nanoparticles in mesoporous silicon: an experimental approach**

Mathieu Lepesant, Benjamin Bardet, Lise-Marie Lacroix, Pierre Fau, Cyril Garnero, Bruno Chaudret, Katerina Soulantica, Thomas Defforge, Damien Valente, Caroline Andreazza, Jérôme Billoué, Patrick Poveda and Gaël Gautier





**Figure S1**: Hysteresis cycle of the bare FeCo NPs recorded at 300K (black line) and 5K(red line) after field cooling the sample under an external field of 3T.





**Figure S2** : Hysteresis cycle of Porous Silicon impregnated with FeCo NPs for 8h (black line), 24h (red line), 72h (blue line) and 7 days (green line).
